# Supplementary material for: Right Vertebral Artery Intermittent Flow Reversal Due to Innominate Artery Dissection
Source: Diagnostics (Basel). 2025 Jun 30;15(13):1668. doi: 10.3390/diagnostics15131668 (PMC12248543; doi:10.3390/diagnostics15131668)
Supplement: Supplementary file 1 [file diagnostics-15-01668-s001.zip › diagnostics-3686416-supplementary.pdf]

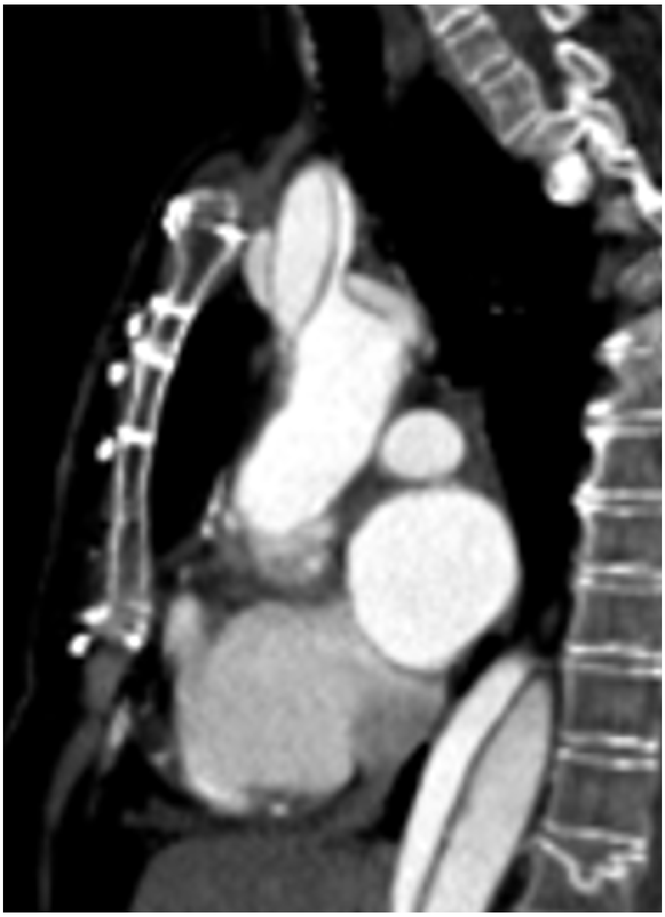

Figure S1: Computed tomography angiography image of type A acute aortic dissection with innominate artery involvement.
